# Targeting diseased tissues by pHLIP insertion at low cell surface pH

**DOI:** 10.3389/fphys.2014.00097

**Published:** 2014-03-13

**Authors:** Oleg A. Andreev, Donald M. Engelman, Yana K. Reshetnyak

**Affiliations:** ^1^Department of Physics, University of Rhode IslandKingston, RI, USA; ^2^Department of Molecular Biophysics and Biochemistry, Yale UniversityNew Haven, CT, USA

**Keywords:** universal health test, imaging, nanotechnology, drug delivery

## Abstract

The discovery of the pH Low Insertion Peptides (pHLIPs®) provides an opportunity to develop imaging and drug delivery agents targeting extracellular acidity. Extracellular acidity is associated with many pathological states, such as those in cancer, ischemic stroke, neurotrauma, infection, lacerations, and others. The metabolism of cells in injured or diseased tissues often results in the acidification of the extracellular environment, so acidosis might be useful as a general marker for the imaging and treatment of diseased states if an effective targeting method can be developed. The molecular mechanism of a pHLIP peptide is based on pH-dependent membrane-associated folding. pHLIPs, being moderately hydrophobic peptides, have high affinities for cellular membranes at normal pH, but fold and insert across membranes at low pH, allowing them to sense pH at the surfaces of cells in diseased tissues, where it is the lowest. Here we discuss the main principles of pHLIP interactions with membrane lipid bilayers at neutral and low pHs, the possibility of tuning the folding and insertion pH by peptide sequence variation, and potential applications of pHLIPs for imaging, therapy and image-guided interventions.

Many diseases such as cancer (solid tumors), ischemia, stroke, infection and others lead to the development of local hypoxia and acidosis. The extracellular acidosis results from enhanced use of glycolysis and production of carbonic and lactic acids, which are intensively pumped out cells to keep intracellular pH near neutral. Acids produced in this way accumulate in extracellular spaces since there is poor blood circulation in diseased tissues. As a consequence, a reversed membrane pH gradient is formed: the extracellular pH (pHe) in diseased tissue is lower than the intracellular pH (pHi) compared with normal tissues (Gerweck and Seetharaman, 1996; Raghunand et al., 1999). Extracellular acidity might serve as a general marker for detecting and targeting diseased tissue. However, such a strategy is challenging, since the bulk extracellular pH in diseased tissue is just 0.5–0.8 pH units lower than the extracellular pH in healthy tissue (Hashim et al., 2011). From a biological standpoint the change is significant, and alters the functions and survival of cells. At the same time, from a chemical standpoint the change is small, so very precise tuning of chemical properties would be needed in a targeting agent. But, an important point that is often overlooked is that the pH is lowest at the surfaces of cells compared to the bulk extracellular pH (Chiche et al., 2010) and increases with distance from membrane, and becoming normal in the vicinity of blood vessels. So, the average pH in tissue is less informative than the pH at cellular surfaces, which might be the main target for the development of pH-sensitive agents. We have been developing a novel class of pH-sensitive delivery agents, pHLIP® s (pH Low Insertion Peptides), which are moderately hydrophobic peptides that can insert into membranes at mild acidic pHs, and which locate themselves at cell surfaces where the pH is lowest (Andreev et al., 2009, 2010a).

## Molecular mechanism of pHLIPs interaction with membrane

**Figure 1:**
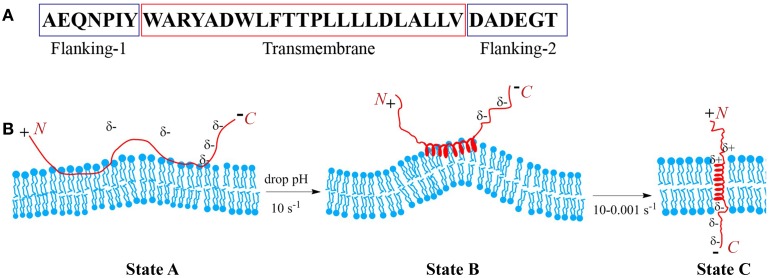
**Schematic presentation of pHLIP interaction with lipid bilayer of membrane**. Sequence of the WT pHLIP **(A)**. At high and neutral pHs pHLIP is associated with the lipid bilayer of membrane. Negative charges of Asp, Glu, and C-terminus prevent partition of the peptide into bilayer. After a drop of the pH, some Asp/Glu residues are protonated, leading to an increase of overall peptide hydrophobicity that triggers deeper partitioning into the bilayer and the formation of an interfacial helix, which results in the distortion of the bilayer. Protonation of Asp/Glu at the inserting end (C-terminus) of the peptide leads to the formation of a transmembrane helix, which reduces the bilayer distortion **(B)**.

Peptides of the pHLIP family consist of flanking and transmembrane (TM) sequences (Figure 1A). The TM part is essential for the interaction with the membrane. The flanking-1 sequence is instrumental for peptide solubility. It usually contains polar and charged residues (Hunt et al., 1997; Reshetnyak et al., 2007; Barrera et al., 2011). The membrane-inserting flanking-2 sequence also can contribute to solubility, and affects the rates of peptide insertion and exit from the membrane (Karabadzhak et al., 2012). In general, peptides of the pHLIP family contain a mixture of natural and/or non-natural amino acids that are hydrophobic and protonatable at low pH. The presence of hydrophobic residues ensures that the peptide maintains an affinity to membrane. The presence of protonatable residues is required (i) for guaranteeing solubility at neutral pH, when they carry negative charges, and (ii) for the enhancement of hydrophobicity at low pH, when the equilibrium is shifted toward protonation.

At neutral and high pH, pHLIP is monomeric and largely unstructured. In the presence of a membrane or lipid bilayer, peptides in aqueous solution coexist with unstructured peptides adsorbed to the surface (Figure 1B). The fraction of the adsorbed peptides is controlled by the lipid:peptide ratio, which in turn affects diffusion of the peptide on membrane surface (Guo and Gai, 2010). Lowering the pH shifts the equilibrium toward folding, membrane insertion, and formation of a TM helix. A subsequent increase of pH promotes the reverse reaction: unfolding of the TM helix and its exit from the bilayer interior. Thus, peptide association with the membrane is distinguishable from the process of peptide partitioning into the bilayer. The latter is accompanied by a coil-helix transition and triggered by a drop in pH. Peptides consisting of L- or D-amino acids show pH-dependent tumor cell targeting *in vitro* and *in vivo* confirming that the mechanism is TM helix formation (right or left handed, respectively), and that it does not depend on any specific recognition event such as binding to a receptor (Andreev et al., 2007; Macholl et al., 2012). The adsorption of pHLIPs to a model membrane surface is accompanied by an energy release of 5–6 kcal/mol, and the insertion process is accompanied by an additional energy release of about 1.8–2.5 kcal/mol. Hence the bilayer affinity of the peptide is 30–50 times higher at low pH than at high pH (Reshetnyak et al., 2008; Weerakkody et al., 2013). The pHLIP insertion results from the protonation of Asp/Glu residues in the TM part of the sequence and its (inserting) flanking-2 end. Carboxyl group protonation leads to an increase in hydrophobicity, which, in turn, triggers TM formation across the hydrophobic bilayer interior (Andreev et al., 2007; Musial-Siwek et al., 2010). Since the surface bound peptide is located at an intermediate zone between polar (aqueous) and non-polar (membrane) environments, the pK for the protonation of Asp and Glu residues is significantly shifted to higher pH values (Harris and Turner, 2002), and the apparent pK of pHLIP insertion can vary from 4.5 to 6.5 (Reshetnyak et al., 2007; Musial-Siwek et al., 2010; Barrera et al., 2011; Weerakkody et al., 2013).

pHLIP insertion is predominantly uni-directional. In most instances it is the C-terminus (flanking-2 end) that propagates across the bilayer and comes out in the cytoplasm (except of the reverse pHLIP sequence with an acetylated N-terminus), while the N-terminus stays in the extracellular region (Reshetnyak et al., 2006; Thevenin et al., 2009). The propagation into the bilayer of the positively charged N-terminal at the flanking-1 end is energetically unfavorable compared to partition of the C-terminal at the flanking-2 end. The latter becomes electrically neutral after the protonation of COO^−^ groups at low pH (Karabadzhak et al., 2012), while the positive charge is difficult to deprotonate and its passage is resisted by the membrane dipole potential. Peptide insertion into the membrane can be sub-divided into two distinct steps: (i) the formation of an interfacial helix and (ii) the movement of the helix across the bilayer to adopt a TM orientation. The timescale for the first process is about 0.1 s, while for the second process it may vary from 0.1 up to 100 s (Andreev et al., 2010b; Karabadzhak et al., 2012), depending on several factors such as (i) the total number of protonatable residues in the sequence, (ii) their pK values, (iii) the presence of protonatable residues and/or polar cargo molecules at the peptide inserting end, and (iv) the compositional properties of the bilayer. The timescale for the peptide to exit from the bilayer varies from several milliseconds to seconds. It is also affected by the number of protonatable residues at the peptide inserting end, especially in the case of insertion into live cells, where the pH in the cytoplasm is 7.2–7.4. The Asp and Glu residues are moved across a bilayer while protonated, and in the cytoplasm they become de-protonated, i.e., negatively charged at pH7.2–7.4 and so serve as anchors for the peptide across a cell membrane, reducing significantly the rate of peptide exit from the bilayer. Thus, the number of protonatable groups on the peptide inserting end slows both insertion and exit rates.

The properties of the lipid bilayer itself play an important role in the process of peptide insertion. At neutral pH, when a pHLIP is unstructured and associated with the outer leaflet of the lipid bilayer, it creates some tension and distortion of the bilayer (Figure 1B). However, due to the fact that the unstructured polypeptide cannot propagate very deep into the bilayer and due to the flexibility of the unstructured polypeptide at the surface of the membrane at high pH, the distortion of the lipid bilayer is not sufficient to render state II, which is thermodynamically stable. However, when the peptide folds and adopts a more rigid, helical structure on the membrane surface (interfacial helical intermediate) the perturbation of the lipids is locally increased. The perturbation favors insertion, since a TM configuration is more compatible with the bilayer.

pHLIP, in contrast to cell-penetrating peptides, stays in the cellular membrane after insertion, translocating one end into the cytoplasm and leaving the other end in the extracellular space. Therefore, the peptide possesses dual delivery capabilities: it can tether cargo molecules to cell surfaces and/or it can inject and release cell-impermeable cargo molecules into cell cytoplasms (Andreev et al., 2009). In the first scenario, a cargo molecule, such as an imaging agent, is attached to the pHLIP's N-terminus, remaining on the cell surface after pHLIP insertion. Transmembrane delivery by pHLIP is based on translocation of polar cargo molecules attached to the C-terminus, using a bond that is stable outside the cell, but cleaved in the cytoplasm. In addition, facilitator or quencher molecules can be attached to the C-terminal part of the peptide together with cargo and/or imaging agents (An et al., 2010; Wijesinghe et al., 2011). The chemical conjugation of various cargo molecules to pHLIPs is straightforward, since Lys and/or Cys residues, as well as other chemical functional moieties, can easily be included in the synthesis of the peptide.

## Targeting of acidic diseased tissue

Cancer cells acquire extensive genetic alterations as they divide in a tumor, including epigenetic regulation sites, point mutations, gene deletions, gene duplications, and chromosomal rearrangements. These changes are heterogeneously distributed within a single tumor (Gillies et al., 2012). The heterogeneity of expression of particular biomarkers at cell surfaces within a tumor and between tumors significantly reduces the effectiveness of agents that target specific biomarkers. On the other hand, low extracellular pH, which is a hallmark of tumors and other pathological states, may provide a target independent of tumor heterogeneity, so agents like pHLIP are worth exploring.

The thermodynamics and kinetics of the pHLIP-membrane interaction predict preferential accumulation in acidic tissues. Indeed, pHLIP peptides conjugated with fluorescent dyes demonstrate excellent *in vivo* targeting of tumors of various origins (Andreev et al., 2007; Reshetnyak et al., 2011), ischemic myocardium (Sosunov et al., 2013), sites of inflammatory arthritis (Andreev et al., 2007), infection (Li et al., 2013) and *ex vivo* staining of cancerous tissue on biopsy samples (Loja et al., 2013). Clinical imaging modalities such as PET (positron emission tomography) and SPECT (single-photon emission computed tomography) also show tumor targeting by pHLIP-based probes (Vavere et al., 2009; Daumar et al., 2012; Macholl et al., 2012). pHLIPs consisting of D-amino acids have the same bilayer interactions as the L-amino acid versions, and show enhanced stability *in vivo*. Targeting of tumor acidity by pHLIP is correlated with MRS (magnetic resonance spectroscopy) measurements of low extracellular pH in tumors on live animals (Vavere et al., 2009). The extracellular acidity in tumors can be modulated by co-injection of glucose (which increases acidity through the Warburg effect) or feeding animals with bicarbonate water (which decreases acidity), resulting in enhanced or reduced pHLIP targeting of tumors, respectively (Vavere et al., 2009; Reshetnyak et al., 2011; Han et al., 2013). Analysis of pHLIP distribution in tumors over time shows that pHLIP can stay in tumors for several days, that tumor borders can be determined with high accuracy and that pHLIP is localized at tumor cell membranes (Segala et al., 2009; Reshetnyak et al., 2011). These properties suggest that fluorescent pHLIP-based agents could be used in image guided resections of tumors during surgery and in analysis of tissue samples.

Adaptation to hypoxia and acidosis may represent key events in the transition from *in situ* to invasive cancer (Gatenby and Gillies, 2007). Metastatic tumors, which have been shown to be more acidic, are labeled more effectively by pHLIP than non-metastatic ones (Reshetnyak et al., 2011). Further, *ex vivo* staining of biopsy samples correlates with stages of tumor development. The samples of normal tissue including those with chronic inflammation are not stained by pHLIP (Loja et al., 2013). Thus, there may be an opportunity to predict tumor invasiveness and distinguish aggressive tumor phenotypes. In addition to the targeting of primary tumors, the targeting of metastatic lesions by pHLIP has been demonstrated, and very small tumors (~1 mm^3^) can be targeted (Reshetnyak et al., 2011). Recently we introduced a family of rationally designed pHLIP variants and demonstrated that tuning by variations of the peptide sequence and, as a result, the physical and chemical properties of peptide-membrane interactions, can modulate tumor targeting, blood clearance, and biodistribution (Weerakkody et al., 2013).

Since acidosis of different magnitudes is a general feature of a number of pathological states, and since the insertion pK of pHLIPs can be adjusted by sequence changes, a “universal health test” might be developed based on imaging by a spectrum of pHLIPs with different pKs. Various diseases might be identified at once and, each suspicious acidic spot could be investigated/diagnosed further.

## pHLIP as a single-molecule transporter

While targeting of diseased tissue is useful for imaging and diagnosis, an exciting use of pHLIP is for treatment using transport of an agent into a cell, where it can reach its cytoplasmic or nuclear target. Most inhibitors and all gene targeting agents are highly polar, and often, are charged molecules with extremely limited plasma membrane permeability. Several different approaches might be employed to move such molecules into a cell:

- modification of a drug molecule to reduce its polarity and enhance membrane-penetration;
- use of various nano-carriers;
- use of cell-penetrating peptides.

In conventional drug design and discovery the Lipinski rules of five (and other, related concepts) are widely used to guide molecular designs. The rules postulate that a successful drug should be hydrophobic and small in order to traverse membranes and reach cytoplasmic targets (e.g., the logarithm of the octanol-water partition coefficient Log*P*_*o/w*_ is −0.4 to +5.6 and the MW is 160 to 480 gmol^−1^) (Lipinski et al., 2001). There are several problems associated with this approach: (i) in some cases, it is difficult or even impossible to convert the polar/charged molecule into a hydrophobic one; (ii) modified drugs may lose their potency; (iii) drugs designed in this way will indiscriminately enter all cells they encounter, reducing effective concentrations in diseased areas and inducing side effects. The side effects can be especially devastating for cancer treatments, since the majority of the drugs are toxic molecules.

Various nanosized drug-delivery vehicles including, but not limited to organic or inorganic nanoparticles, liposomes, micelles, viral particles, polymers, dendrimers, and others, have been designed for complexation or encapsulation of polar/charged therapeutic molecules. Nano-carriers usually are multifunctional, and targeting, imaging and therapeutic molecules can be combined in a single nano-carrier (Ferrari, 2005; Davis et al., 2008; Gindy and Prud'homme, 2009; Cheng et al., 2012; Han et al., 2013). Some targeting of vascularized tumors can result from the enhanced permeability of tumor vessels, resulting in preferential nanoparticle retention. Despite the advantages, there are significant limitations related to the use of nano-sized carriers, such as complexity and difficulty in manufacturing, reduced stability, potential toxicity of some nanomaterials, and reduced tumor-penetration ability. Significantly, all nanocarriers enter cells via one of the endocytotic routes, which are usually of low efficacy due to the entrapment of therapeutic molecule in the endosomal/lysosomal compartments.

Cell-penetrating, positively-charged peptides such as TAT [a peptide derived from the trans-activating transcriptional activator (TAT) protein], antennapedia, arginine-rich peptides and others, promote enhanced cellular uptake of molecules attached to them. The majority of cell-penetrating peptides enter cells via one of the endocytotic routes (Bechara and Sagan, 2013), although a number of reports indicate the possibility of direct diffusion across the membrane lipid bilayer. Commonly in these cases a hydrophobic motif is added to the peptide (Carrigan and Imperiali, 2005; Takayama et al., 2009), which, unfortunately, increases the probability of membrane destabilization and toxicity (Chan et al., 2006). Furthermore, cell-penetrating peptides are positively charged, which is also associated with the enhanced toxicity, limiting their use. Microbial peptides induce permeabilization of the plasma membranes and act cooperatively, which is non-advantageous for drug delivery systems. In each of these cases, there is no preferential tumor targeting, since the peptides enter both diseased and normal cells.

The folding and insertion into membrane of constitutive membrane proteins is facilitated by complex molecular machines *in vivo*, including the translocon that places most transmembrane helices across the bilayer (Van Den Berg et al., 2004; White and Von Heijne, 2004; Osborne et al., 2005). However, non-constitutive membrane proteins can insert transmembrane helices (TMs) into a lipid bilayer without assistance. Because the spontaneous insertion and folding of a TM peptide into a lipid bilayer approaches a free energy minimum, an insertion event is accompanied by the release of energy. The moderately hydrophobic TM forming peptides such pHLIPs might be considered as the basis for a novel class of delivery agents: single-molecular transporters. The membrane-associated folding of pHLIPs is accompanied by the release of about 2 kcal/mol of energy, which could be used as a biasing potential to favor the movement of polar, cell-impermeable cargo molecules across membrane into a cytoplasm. A significant feature of the process of peptide folding into a membrane is that it ensures a much higher cooperativity of the transition compared to the simple pH-driven diffusion of weak bases across a membrane. Translocation is selective for low pH, and various types of cargo molecules attached by disulfides to the inserting end of pHLIP (in most cases the C-terminus) can be transported into a cell and released in cytoplasm. We have demonstrated that pHLIP can translocate fluorescent dyes, cyclic peptides, polar toxins and peptide nucleic acids into cancer cells, and the properties of translocatable molecules can significantly violate Lipinski's rules, being larger and much more polar (Reshetnyak et al., 2006; Thevenin et al., 2009; An et al., 2010; Wijesinghe et al., 2011; Moshnikova et al., 2013). The translocation is fast (seconds to minutes), pH- and concentration-dependent and can be modulated by tuning of the hydrophobicity of the pHLIP inserting end. Compounds such as phalloidin and α-amanitin have not been considered as anti-cancer therapeutic agents, since they cannot cross cellular membranes (Log*P*_*o/w*_ is about −1.5 and the MW is about 800–1000 gmol^−1^). These toxins are selectively taken up by hepatocytes, leading to liver damage, since hepatocytes possess special transporting systems to translocate small cyclic molecules (Magdalan et al., 2010; Santi et al., 2012). Recently, it has been demonstrated that amanitin conjugated with an anti-human epithelial cell adhesion antibody induces inhibition of cell proliferation and suppression of pancreatic tumor growth (Moldenhauer et al., 2012). Conjugation of phallo- and amanita toxins to the pHLIP's inserting end via cleavable bond also leads to cell death in a low extracellular pH environment. The major mechanism of polar cargo delivery is direct translocation across the plasma membrane by pHLIP and cleavage of S-S bond in the reducing environment of the cytoplasm (other types of bonds might be used). Amanitin conjugated to pHLIP via non-cleavable cross-linkers or attached to the non-inserting end of the peptide was not able to induce cell death.

## pHLIP-mediated delivery of nanoparticles and liposomes

The pHLIP technology can also substantially improve the delivery of nanoparticles and liposomes to acidic diseased tissue (Davies et al., 2012; Sosunov et al., 2013; Wijesinghe et al., 2013; Yao et al., 2013a,b). The intratumoral and intravenous administration of gold nanoparticles conjugated with pHLIP both demonstrated 6-fold enhancement of tumor uptake compared to gold nanoparticles with no pHLIP (Yao et al., 2013a). Statistically significant reduction of gold accumulation was observed in acidic tumors and kidneys when pH-nonsensitive K-pHLIP (where Asp residues are replaced by Lys) was used as a vehicle, suggesting an important role of pH in the pHLIP-mediated targeting of gold nanoparticles. Gold nanoparticles delivered to a tumor might be used for local enhancement of radiation therapy.

We have also developed pH-sensitive, “fusogenic” pHLIP-coated liposomes (Wijesinghe et al., 2013; Yao et al., 2013b). The presence of pHLIP on the surfaces of liposomes enhances membrane fusion and lipid exchange in a pH dependent fashion, leading to an increase of cellular uptake and payload release, and consequent inhibition of cell proliferation by liposomes containing ceramide. Using two murine ischemia models we show that pHLIP-coated liposomes bind acidic ischemic but not normal regions of myocardium, while liposomes coated with PEG showed no preference in targeting of acidic ischemic tissue (Sosunov et al., 2013).

pHLIP-coated liposomes have been used for selective delivery of gramicidin nano-pores to cancer cells (Wijesinghe et al., 2013). Gramicidin channels inserted into the cancer cell membranes allow a flux of protons into the cytoplasm, and also disrupt the transmembrane balance of other monovalent cations, which induces cell apoptosis. Selective incorporation of functional gramicidin channels opens an opportunity for the delivery of other membrane peptides and proteins, which might find wide application in biotechnology and medicine.

**Figure 2:**
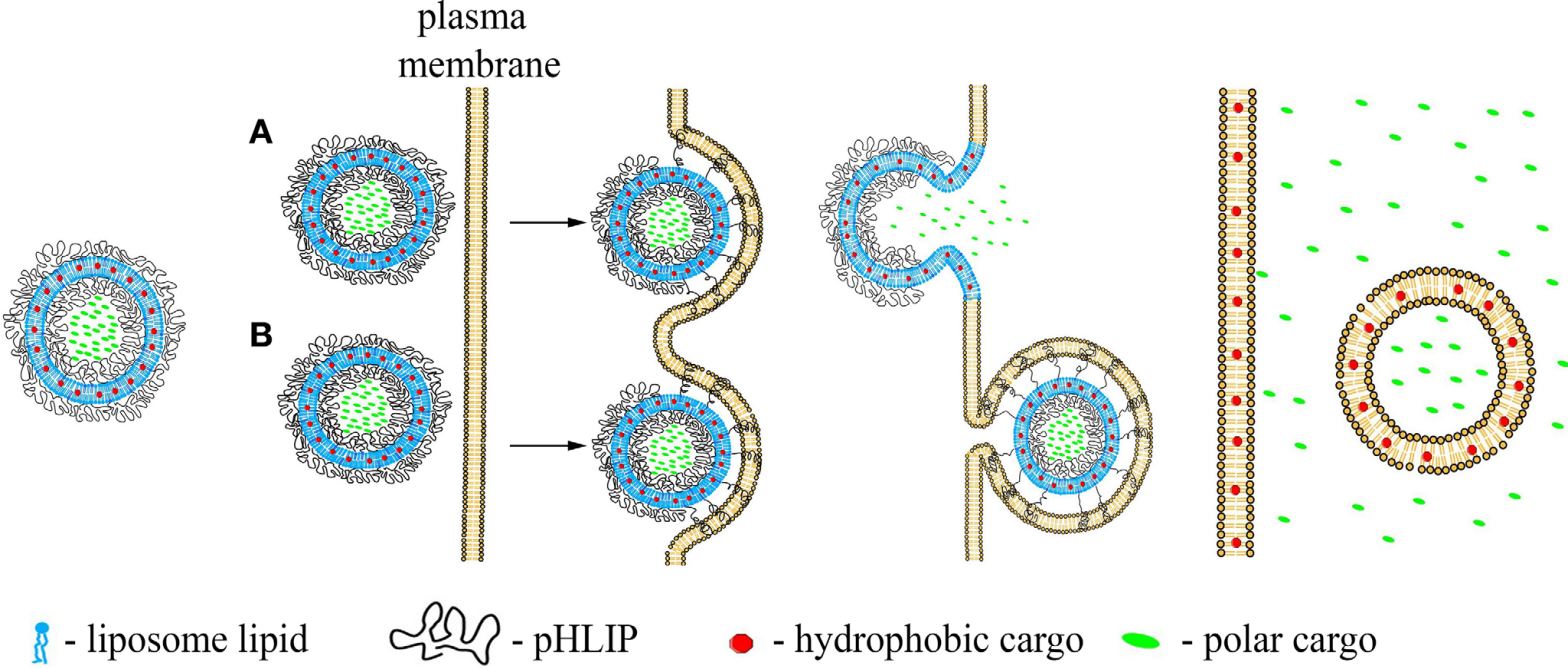
**Schematic presentation of pHLIP-coated liposome entry into a cell**. pHLIP coated Liposomes with encapsulated polar cargo molecules or carrying hydrophobic cargoes in their bilayers can deliver payloads to cell membranes and cytoplasms. The acidity of diseased tissue results in pHLIP-mediated fusion of liposomes with plasma membranes at low extracellular pH **(A)** or fusion with endosomal membranes **(B)**.

In contrast to the pHLIP action as a single-molecule transporter when a single cargo molecule is attached to the single pHLIP peptide, multiple pHLIPs on the surfaces of liposomes or lipid-coated nanoparticles will interact with cellular membranes cooperatively, promoting membrane deformation, and tension. This binding will lead to lipid exchange, enhanced cellular uptake and/or direct fusion accompanied by the delivery of hydrophobic molecules to cellular membranes and polar cargo molecules to cytoplasm (Figures 2A,B). These processes are initiated in the acidic environment of diseased tissue, or, in the case of endocytotic uptake, will be even more effectively promoted by the low pH of endosomes/lysosomes, leading to the cytoplasmic release of cargo.

## Concluding remarks

Peptides of pHLIP family can be used to target cells diseased tissues as a result of extracellular acidity. Tuning the properties of pHLIP by sequence variation allows alteration of pharmacokinetics and targeting ability. pHLIPs conjugated with cell-impermeable cargoes are a novel class of delivery agents: single-molecule transporters for direct cytoplasmic delivery of polar cargo molecules into cells in acidic, diseased tissues. Multiple pHLIPs on the surfaces of nano-sized particles promote pH-mediated distortion of cellular membranes, leading to the enhanced delivery of payloads to cytosols or cellular membranes.

### Conflict of interest statement

The authors declare that the research was conducted in the absence of any commercial or financial relationships that could be construed as a potential conflict of interest.

## References

[B1] An M. Wijesinghe D. Andreev O. A. Reshetnyak Y. K. Engelman D. M. (2010). pH-(low)-insertion-peptide (pHLIP) translocation of membrane impermeable phalloidin toxin inhibits cancer cell proliferation. Proc. Natl. Acad. Sci. U.S.A. 107, 20246–20250 10.1073/pnas.1014403107 21048084 PMC2996653

[B2] Andreev O. A. Dupuy A. D. Segala M. Sandugu S. Serra D. A. Chichester C. O. (2007). Mechanism and uses of a membrane peptide that targets tumors and other acidic tissues *in vivo*. Proc. Natl. Acad. Sci. U.S.A. 104, 7893–7898 10.1073/pnas.0702439104 17483464 PMC1861852

[B3] Andreev O. A. Engelman D. M. Reshetnyak Y. K. (2009). Targeting acidic diseased tissue: new technology based on use of the pH (Low) Insertion Peptide (pHLIP). Chim. Oggi 27, 34–37 20037661 PMC2796806

[B4] Andreev O. A. Engelman D. M. Reshetnyak Y. K. (2010a). pH-sensitive membrane peptides (pHLIPs) as a novel class of delivery agents. Mol. Membr. Biol. 27, 341–352 10.3109/09687688.2010.509285 20939768 PMC4691209

[B5] Andreev O. A. Karabadzhak A. G. Weerakkody D. Andreev G. O. Engelman D. M. Reshetnyak Y. K. (2010b). pH (low) insertion peptide (pHLIP) inserts across a lipid bilayer as a helix and exits by a different path. Proc. Natl. Acad. Sci. U.S.A. 107, 4081–4086 10.1073/pnas.0914330107 20160113 PMC2840156

[B6] Barrera F. N. Weerakkody D. Anderson M. Andreev O. A. Reshetnyak Y. K. Engelman D. M. (2011). Roles of carboxyl groups in the transmembrane insertion of peptides. J. Mol. Biol. 413, 359–371 10.1016/j.jmb.2011.08.010 21888917 PMC3193594

[B7] Bechara C. Sagan S. (2013). Cell-penetrating peptides: 20 years later, where do we stand? FEBS Lett. 587, 1693–1702 10.1016/j.febslet.2013.04.031 23669356

[B8] Carrigan C. N. Imperiali B. (2005). The engineering of membrane-permeable peptides. Anal. Biochem. 341, 290–298 10.1016/j.ab.2005.03.026 15907875

[B9] Chan D. I. Prenner E. J. Vogel H. J. (2006). Tryptophan- and arginine-rich antimicrobial peptides: structures and mechanisms of action. Biochim. Biophys. Acta 1758, 1184–1202 10.1016/j.bbamem.2006.04.006 16756942

[B10] Cheng Z. Al Zaki A. Hui J. Z. Muzykantov V. R. Tsourkas A. (2012). Multifunctional nanoparticles: cost versus benefit of adding targeting and imaging capabilities. Science 338, 903–910 10.1126/science.1226338 23161990 PMC3660151

[B11] Chiche J. Brahimi-Horn M. C. Pouyssegur J. (2010). Tumour hypoxia induces a metabolic shift causing acidosis: a common feature in cancer. J. Cell. Mol. Med. 14, 771–794 10.1111/j.1582-4934.2009.00994.x 20015196 PMC3823111

[B12] Daumar P. Wanger-Baumann C. A. Pillarsetty N. Fabrizio L. Carlin S. D. Andreev O. A. (2012). Efficient (18)F-labeling of large 37-amino-acid pHLIP peptide analogues and their biological evaluation. Bioconjug. Chem. 23, 1557–1566 10.1021/bc3000222 22784215 PMC3529145

[B13] Davies A. Lewis D. J. Watson S. P. Thomas S. G. Pikramenou Z. (2012). pH-controlled delivery of luminescent europium coated nanoparticles into platelets. Proc. Natl. Acad. Sci. U.S.A. 109, 1862–1867 10.1073/pnas.1112132109 22308346 PMC3277523

[B14] Davis M. E. Chen Z. G. Shin D. M. (2008). Nanoparticle therapeutics: an emerging treatment modality for cancer. Nat. Rev. Drug Discov. 7, 771–782 10.1038/nrd2614 18758474

[B15] Ferrari M. (2005). Cancer nanotechnology: opportunities and challenges. Nat. Rev. Cancer 5, 161–171 10.1038/nrc1566 15738981

[B16] Gatenby R. A. Gillies R. J. (2007). Glycolysis in cancer: a potential target for therapy. Int. J. Biochem. Cell Biol. 39, 1358–1366 10.1016/j.biocel.2007.03.021 17499003

[B17] Gerweck L. E. Seetharaman K. (1996). Cellular pH gradient in tumor versus normal tissue: potential exploitation for the treatment of cancer. Cancer Res. 56, 1194–1198 8640796

[B18] Gillies R. J. Verduzco D. Gatenby R. A. (2012). Evolutionary dynamics of carcinogenesis and why targeted therapy does not work. Nat. Rev. Cancer 12, 487–493 10.1038/nrc3298 22695393 PMC4122506

[B19] Gindy M. E. Prud'homme R. K. (2009). Multifunctional nanoparticles for imaging, delivery and targeting in cancer therapy. Expert Opin. Drug Deliv. 6, 865–878 10.1517/17425240902932908 19637974

[B20] Guo L. Gai F. (2010). Heterogeneous diffusion of a membrane-bound pHLIP peptide. Biophys. J. 98, 2914–2922 10.1016/j.bpj.2010.03.050 20550904 PMC2884227

[B21] Han L. Ma H. Guo Y. Kuang Y. He X. Jiang C. (2013). pH-controlled delivery of nanoparticles into tumor cells. Adv. Healthc. Mater. 2, 1435–1439 10.1002/adhm.201300013 23564477

[B22] Harris T. K. Turner G. J. (2002). Structural basis of perturbed pKa values of catalytic groups in enzyme active sites. IUBMB Life 53, 85–98 10.1080/15216540211468 12049200

[B23] Hashim A. I. Zhang X. Wojtkowiak J. W. Martinez G. V. Gillies R. J. (2011). Imaging pH and metastasis. NMR Biomed. 24, 582–591 10.1002/nbm.1644 21387439 PMC3740268

[B24] Hunt J. F. Earnest T. N. Bousche O. Kalghatgi K. Reilly K. Horvath C. (1997). A biophysical study of integral membrane protein folding. Biochemistry 36, 15156–15176 10.1021/bi970146j 9398244

[B25] Karabadzhak A. G. Weerakkody D. Wijesinghe D. Thakur M. S. Engelman D. M. Andreev O. A. (2012). Modulation of the pHLIP transmembrane helix insertion pathway. Biophys. J. 102, 1846–1855 10.1016/j.bpj.2012.03.021 22768940 PMC3328699

[B26] Li N. Yin L. Thevenin D. Yamada Y. Limmon G. Chen J. (2013). Peptide targeting and imaging of damaged lung tissue in influenza-infected mice. Future Microbiol. 8, 257–269 10.2217/fmb.12.134 23374130 PMC3677567

[B27] Lipinski C. A. Lombardo F. Dominy B. W. Feeney P. J. (2001). Experimental and computational approaches to estimate solubility and permeability in drug discovery and development settings. Adv. Drug Deliv. Rev. 46, 3–26 10.1016/S0169-409X(00)00129-0 11259830

[B28] Loja M. N. Luo Z. Greg Farwell D. Luu Q. C. Donald P. J. Amott D. (2013). Optical molecular imaging detects changes in extracellular pH with the development of head and neck cancer. Int. J. Cancer 132, 1613–1623 10.1002/ijc.27837 22965462 PMC4405778

[B29] Macholl S. Morrison M. S. Iveson P. Arbo B. E. Andreev O. A. Reshetnyak Y. K. (2012). *In vivo* pH imaging with (99m)Tc-pHLIP. Mol. Imaging Biol. 14, 725–734 10.1007/s11307-012-0549-z 22371188 PMC3909815

[B30] Magdalan J. Ostrowska A. Piotrowska A. Izykowska I. Nowak M. Gomulkiewicz A. (2010). alpha-Amanitin induced apoptosis in primary cultured dog hepatocytes. Folia Histochem. Cytobiol. 48, 58–62 10.2478/v10042-010-0010-6 20529816

[B31] Moldenhauer G. Salnikov A. V. Luttgau S. Herr I. Anderl J. Faulstich H. (2012). Therapeutic potential of amanitin-conjugated anti-epithelial cell adhesion molecule monoclonal antibody against pancreatic carcinoma. J. Natl. Cancer Inst. 104, 622–634 10.1093/jnci/djs140 22457476

[B32] Moshnikova A. Moshnikova V. Andreev O. A. Reshetnyak Y. K. (2013). Antiproliferative effect of pHLIP-amanitin. Biochemistry 52, 1171–1178 10.1021/bi301647y 23360641 PMC3580847

[B33] Musial-Siwek M. Karabadzhak A. Andreev O. A. Reshetnyak Y. K. Engelman D. M. (2010). Tuning the insertion properties of pHLIP. Biochim. Biophys. Acta 1798, 1041–1046 10.1016/j.bbamem.2009.08.023 19766589 PMC2862812

[B34] Osborne A. R. Rapoport T. A. Van Den Berg B. (2005). Protein translocation by the Sec61/SecY channel. Annu. Rev. Cell Dev. Biol. 21, 529–550 10.1146/annurev.cellbio.21.012704.133214 16212506

[B35] Raghunand N. Altbach M. I. Van Sluis R. Baggett B. Taylor C. W. Bhujwalla Z. M. (1999). Plasmalemmal pH-gradients in drug-sensitive and drug-resistant MCF-7 human breast carcinoma xenografts measured by 31P magnetic resonance spectroscopy. Biochem. Pharmacol. 57, 309–312 10.1016/S0006-2952(98)00306-2 9890558

[B36] Reshetnyak Y. K. Andreev O. A. Lehnert U. Engelman D. M. (2006). Translocation of molecules into cells by pH-dependent insertion of a transmembrane helix. Proc. Natl. Acad. Sci. U.S.A. 103, 6460–6465 10.1073/pnas.0601463103 16608910 PMC1435408

[B37] Reshetnyak Y. K. Andreev O. A. Segala M. Markin V. S. Engelman D. M. (2008). Energetics of peptide (pHLIP) binding to and folding across a lipid bilayer membrane. Proc. Natl. Acad. Sci. U.S.A. 105, 15340–15345 10.1073/pnas.0804746105 18829441 PMC2556629

[B38] Reshetnyak Y. K. Segala M. Andreev O. A. Engelman D. M. (2007). A monomeric membrane peptide that lives in three worlds: in solution, attached to, and inserted across lipid bilayers. Biophys. J. 93, 2363–2372 10.1529/biophysj.107.109967 17557792 PMC1965453

[B39] Reshetnyak Y. K. Yao L. Zheng S. Kuznetsov S. Engelman D. M. Andreev O. A. (2011). Measuring tumor aggressiveness and targeting metastatic lesions with fluorescent pHLIP. Mol. Imaging Biol. 13, 1146–1156 10.1007/s11307-010-0457-z 21181501 PMC3227673

[B40] Santi L. Maggioli C. Mastroroberto M. Tufoni M. Napoli L. Caraceni P. (2012). Acute liver failure caused by amanita phalloides poisoning. Int. J. Hepatol. 2012, 487480 10.1155/2012/487480 22811920 PMC3395149

[B41] Segala J. Engelman D. M. Reshetnyak Y. K. Andreev O. A. (2009). Accurate analysis of tumor margins using a fluorescent ph low insertion peptide (pHLIP). Int. J. Mol. Sci. 10, 3478–3487 10.3390/ijms10083478 20111691 PMC2812834

[B42] Sosunov E. A. Anyukhovsky E. P. Sosunov A. A. Moshnikova A. Wijesinghe D. Engelman D. M. (2013). pH (low) insertion peptide (pHLIP) targets ischemic myocardium. Proc. Natl. Acad. Sci. U.S.A. 110, 82–86 10.1073/pnas.1220038110 23248283 PMC3538213

[B43] Takayama K. Nakase I. Michiue H. Takeuchi T. Tomizawa K. Matsui H. (2009). Enhanced intracellular delivery using arginine-rich peptides by the addition of penetration accelerating sequences (Pas). J. Control. Release 138, 128–133 10.1016/j.jconrel.2009.05.019 19465072

[B44] Thevenin D. An M. Engelman D. M. (2009). pHLIP-mediated translocation of membrane-impermeable molecules into cells. Chem. Biol. 16, 754–762 10.1016/j.chembiol.2009.06.006 19635412 PMC2741147

[B45] Van Den Berg B. Clemons W. M. Jr. Collinson I. Modis Y. Hartmann E. Harrison S. C. (2004). X-ray structure of a protein-conducting channel. Nature 427, 36–44 10.1038/nature02218 14661030

[B46] Vavere A. L. Biddlecombe G. B. Spees W. M. Garbow J. R. Wijesinghe D. Andreev O. A. (2009). A novel technology for the imaging of acidic prostate tumors by positron emission tomography. Cancer Res. 69, 4510–4516 10.1158/0008-5472.CAN-08-3781 19417132 PMC2690701

[B47] Weerakkody D. Moshnikova A. Thakur M. S. Moshnikova V. Daniels J. Engelman D. M. (2013). Family of pH (low) insertion peptides for tumor targeting. Proc. Natl. Acad. Sci. U.S.A. 110, 5834–5839 10.1073/pnas.1303708110 23530249 PMC3625278

[B48] White S. H. Von Heijne G. (2004). The machinery of membrane protein assembly. Curr. Opin. Struct. Biol. 14, 397–404 10.1016/j.sbi.2004.07.003 15313232

[B49] Wijesinghe D. Arachchige M. C. Lu A. Reshetnyak Y. K. Andreev O. A. (2013). pH dependent transfer of nano-pores into membrane of cancer cells to induce apoptosis. Sci. Rep. 3:3560 10.1038/srep03560 24356337 PMC3868956

[B50] Wijesinghe D. Engelman D. M. Andreev O. A. Reshetnyak Y. K. (2011). Tuning a polar molecule for selective cytoplasmic delivery by a pH (Low) insertion peptide. Biochemistry 50, 10215–10222 10.1021/bi2009773 22029270 PMC3229090

[B51] Yao L. Daniels J. Moshnikova A. Kuznetsov S. Ahmed A. Engelman D. M. (2013a). pHLIP peptide targets nanogold particles to tumors. Proc. Natl. Acad. Sci. U.S.A. 110, 465–470 10.1073/pnas.1219665110 23267062 PMC3545779

[B52] Yao L. Daniels J. Wijesinghe D. Andreev O. A. Reshetnyak Y. K. (2013b). pHLIP(R)-mediated delivery of PEGylated liposomes to cancer cells. J. Control. Release 167, 228–237 10.1016/j.jconrel.2013.01.037 23416366 PMC3630259

